# Barriers to Lifestyle Modification and Willingness to Join Weight Loss Programs Among Overweight or Obese Adults in Saudi Family Medicine Clinics

**DOI:** 10.7759/cureus.86223

**Published:** 2025-06-17

**Authors:** Arwa M Alshaikh, Alshaymaa A Alshaikh, Hind S Alatawi, Jood A Alshaikh, Osama M Alshaikh, Mohd H Yusuf

**Affiliations:** 1 Family Medicine, Jeddah First Health Cluster, Jeddah, SAU; 2 Family Medicine, National Guard Hospital, Jeddah, SAU; 3 Family Medicine, King Fahad Armed Forces Hospital, Jeddah, SAU; 4 Family Medicine, King Abdulaziz University, Jeddah, SAU; 5 General Practice, Salmaniya Medical Complex, Manama, BHR

**Keywords:** barriers, family medicine, lifestyle modification, obesity, overweight, physician engagement, primary care, saudi arabia, structured weight loss programs, weight loss attempts

## Abstract

Background

Obesity is a growing public health concern in Saudi Arabia, with increasing prevalence and related comorbidities. Lifestyle modification is key to treatment, yet patient-level barriers and readiness to engage in structured weight management programs remain underexplored in primary care.

Objective

The aim of this study was to examine perceived barriers to lifestyle change, past weight loss attempts, and factors associated with willingness to join structured weight loss programs among overweight or obese adults attending family medicine clinics in Saudi Arabia.

Methods

A cross-sectional study was conducted from March to May 2025 at four urban family medicine clinics. Adults aged ≥18 years with a BMI of ≥25 kg/m² were consecutively recruited. Participants completed a questionnaire on demographics, weight loss attempts, barriers, information sources, and willingness to participate in structured programs. Bivariate analyses and multivariable logistic regression identified factors associated with weight loss attempts and willingness to join programs.

Results

Among 312 participants, 199 (63.8%) were female. Most were aged 30-49 years (167 [53.5%]). Weight loss attempts in the past year were reported by 213 (68.3%). Common barriers included lack of motivation (219 [70.2%]), limited time to exercise (213 [68.3%]), and cost of healthy food (205 [65.7%]). Only 138 (44.2%) recalled physician counseling on weight management. In bivariate analyses, female sex (147 of 199 women [73.9%] vs. 66 of 113 men [58.4%], P=0.004) and university/postgraduate education (139 of 181 [76.8%] vs. 74 of 131 [56.5%], P<0.001) were associated with weight loss attempts. Multivariable logistic regression showed female sex (adjusted OR 1.62; 95% CI, 1.01-2.60; P=0.045), university/postgraduate education (adjusted OR 2.10; 95% CI, 1.28-3.46; P=0.003), and identifying a physician as the main information source (adjusted OR 2.44; 95% CI, 1.47-4.06; P<0.001) were independently associated with willingness to join a structured weight loss program.

Conclusions

Most overweight or obese adults attending family clinics in Saudi Arabia have attempted weight loss but face multiple barriers, including motivation, time, and cost. Physician engagement and higher education levels increase readiness to participate in structured programs. Tailored interventions addressing these barriers are critical to improving obesity management in primary care.

## Introduction

Obesity is a major public health challenge worldwide, with rising prevalence in both high- and middle-income countries. In Saudi Arabia, recent national surveys estimate that more than one-third of adults have obesity, a rate that has more than doubled over the past two decades [1-3]. This trend has been accompanied by a sharp increase in obesity-related comorbidities, including type 2 diabetes, hypertension, and cardiovascular disease. Despite widespread recognition of the problem, effective and sustainable weight management remains elusive for many patients [4,5].

Lifestyle modification - encompassing changes in diet, physical activity, and behavior - is the cornerstone of obesity treatment and prevention [6,7]. Clinical guidelines from leading health authorities recommend that primary care physicians provide regular counseling, support goal setting, and refer patients to structured weight management programs when appropriate. However, the implementation of these guidelines in real-world primary care settings has been inconsistent, with barriers reported at both the provider and patient levels [7-9].

Patients often encounter multiple obstacles that limit their ability to initiate and sustain lifestyle changes. These barriers may include a lack of motivation, low health literacy, limited time or financial resources, competing family responsibilities, and inadequate support from healthcare providers [10-13]. In the Middle East, cultural norms, gender roles, and the built environment may further influence patients' readiness and ability to adopt healthier lifestyles. Despite these challenges, there is a paucity of region-specific data describing the barriers faced by patients with obesity, particularly in primary care settings where most chronic disease management occurs [2,8].

Understanding these barriers is essential for designing effective, culturally tailored interventions that address the needs and constraints of patients. This study aimed to explore the perceived barriers to lifestyle modification among overweight or obese adults who attend family medicine clinics in Saudi Arabia and to identify factors associated with past weight loss attempts and willingness to participate in structured weight management programs.

## Materials and methods

Study design and setting

We conducted a cross-sectional study using a structured questionnaire to assess barriers to lifestyle modification among overweight or obese adult patients attending family medicine clinics in Saudi Arabia. This cross-sectional study was conducted between March and May 2025 at the Ministry of Health primary health care clinics located in the Eastern Province of Saudi Arabia. The study was approved by the Research Ethics Committee (Reference No. REC-2024-18821) on December 10, 2024, and conducted according to the principles of the Declaration of Helsinki.

Participants

Eligible participants were adults aged 18 years or older with a BMI of ≥25 kg/m², based on recent clinical measurements or self-report. Patients were recruited consecutively in waiting areas and invited to complete the questionnaire voluntarily. Those who were pregnant, unable to complete the questionnaire due to cognitive or language barriers, or previously enrolled were excluded. All participants provided verbal informed consent before participation.

Questionnaire development

The questionnaire was developed based on a review of prior literature and expert input from family physicians, endocrinologists, and public health specialists. It consisted entirely of multiple-choice questions and included five sections: sociodemographic and clinical characteristics, past attempts at weight loss, perceived barriers to lifestyle change, sources of weight-related information, and willingness to participate in structured programs. A pilot version was tested on 20 patients to ensure clarity and relevance; minor revisions were made accordingly (Appendix A). Data from the pilot was not included in the final analysis.

Data collection

Trained research assistants administered paper-based questionnaires in waiting areas and assisted participants when needed. To ensure confidentiality, no personal identifiers were collected. Each questionnaire was checked for completeness before inclusion. Completed forms were entered into a secure database by two independent data entry personnel to minimize transcription errors.

Outcome measures

The primary outcome was the proportion of participants who reported having attempted to lose weight within the past 12 months. Secondary outcomes included perceived barriers to lifestyle change and willingness to join a structured weight loss program.

Statistical analysis

Descriptive statistics were used to summarize participant characteristics and response patterns. Categorical variables were presented as frequencies and percentages. Bivariate associations between participant characteristics and weight loss attempts were assessed using the chi-square test. Multivariable logistic regression was performed to identify independent predictors of willingness to join a structured weight loss program. Covariates included sex, age group, educational level, comorbid conditions, and key barriers. Adjusted odds ratios and 95% confidence intervals were reported. All analyses were conducted using SPSS Statistics Version 26 (IBM Corp., Armonk, NY). A two-sided P-value of less than 0.05 was considered statistically significant.

## Results

Participant characteristics

A total of 312 overweight or obese adult patients completed the questionnaire in family medicine clinics across Saudi Arabia. Most participants were between 30 and 49 years of age: 78 (25.0%) were aged 30-39 years, and 89 (28.5%) were aged 40-49 years; 61 participants (19.6%) were aged 50-59 years, 41 (13.1%) were aged 18-29 years, and 43 (13.8%) were aged 60 years or older (Table 1). A total of 199 (63.8%) participants were female. The most common comorbid condition was diabetes mellitus, reported by 142 (45.5%) participants, followed by hypertension in 129 (41.3%) and dyslipidemia in 103 (33.0%). Regarding educational level, 181 (58.0%) participants had completed university or postgraduate education. Most participants were employed (147 [47.1%]).

**Table 1:** Characteristics of the study population (N=312) This table presents the demographic and clinical characteristics of 312 overweight or obese adult patients attending family medicine clinics in Saudi Arabia. Percentages are based on the total number of patients unless otherwise specified. †Multiple responses allowed.

Characteristic		No. of patients (%)
Age group (years)	18–29	41 (13.1%)
	30–39	78 (25.0%)
	40–49	89 (28.5%)
	50–59	61 (19.6%)
	≥60	43 (13.8%)
Sex	Male	113 (36.2%)
	Female	199 (63.8%)
Marital status	Single	58 (18.6%)
	Married	223 (71.5%)
	Divorced	17 (5.4%)
	Widowed	14 (4.5%)
Education level	No formal education	16 (5.1%)
	Primary school	31 (9.9%)
	High school	84 (26.9%)
	University degree	138 (44.2%)
	Postgraduate degree	43 (13.8%)
Employment status	Employed	147 (47.1%)
	Unemployed	76 (24.4%)
	Retired	28 (9.0%)
	Student	12 (3.8%)
	Homemaker	49 (15.7%)
Chronic diseases†	None	79 (25.3%)
	Diabetes mellitus	142 (45.5%)
	Hypertension	129 (41.3%)
	Dyslipidemia	103 (33.0%)
	Hypothyroidism	37 (11.9%)
	Other	19 (6.1%)

Attempts at weight loss

Of the 312 participants, 213 (68.3%) reported that they had attempted to lose weight in the past year (Table 2). The most commonly reported strategies included reducing portion sizes (173 participants [55.4%]), increasing physical activity (124 [39.7%]), reducing intake of sugary drinks (not specified in Table 2), and eating out less frequently (data not shown). Fewer participants reported trying intermittent fasting (83 [26.6%]) or calorie counting (64 [20.5%]). Only 48 (15.4%) participants had tried commercial weight loss products, and 40 (12.8%) had joined a structured weight loss program or clinic.

**Table 2:** Previous weight loss attempts and information sources (N=312) This table summarizes the duration since obesity diagnosis, weight loss attempts in the past year, methods used for weight loss, and main sources of weight loss information among 312 adult patients attending family medicine clinics in Saudi Arabia. Percentages are based on the total number of patients unless otherwise specified. †Multiple responses allowed.

Variable		No. of patients (%)
Duration since obesity diagnosis	<1 year	47 (15.1%)
	1–2 years	66 (21.2%)
	3–5 years	102 (32.7%)
	>5 years	97 (31.1%)
Tried to lose weight in the past year	Yes	213 (68.3%)
	No	99 (31.7%)
Methods tried for weight loss†	Diet changes	173 (55.4%)
	Exercise	124 (39.7%)
	Weight loss medications	62 (19.9%)
	Herbal/traditional remedies	47 (15.1%)
	Bariatric surgery	21 (6.7%)
	None	79 (25.3%)
Main source of weight loss information	Physician	102 (32.7%)
	Social media	84 (26.9%)
	Family/friends	56 (17.9%)
	TV or radio	29 (9.3%)
	Online articles	33 (10.6%)
	I do not seek information	8 (2.6%)

Perceived barriers to lifestyle modification

The most commonly reported barrier to making lifestyle changes was lack of motivation, with 219 (70.2%) participants agreeing or strongly agreeing to this barrier (131 [42.0%] strongly agree; 88 [28.2%] agree) (Table 3). Limited time to exercise was reported by 213 (68.3%) participants, and 205 (65.7%) felt that healthy food options were too expensive. Other frequently reported barriers included family or cultural obligations (not explicitly reported in Table 3), lack of safe places to exercise (138 [44.3%]), and insufficient family support (124 [39.7%]). A smaller number of participants (42 [13.5%]) indicated that they did not know how to begin making lifestyle changes.

**Table 3:** Agreement with reported barriers to lifestyle modification (N=312) This table presents the levels of agreement among 312 overweight or obese patients regarding common barriers to lifestyle modification. Data are expressed as number of patients (%) for each response category.

Barrier	Strongly agree	Agree	Neutral	Disagree	Strongly disagree
Lack of time to exercise	117 (37.5%)	96 (30.8%)	44 (14.1%)	34 (10.9%)	21 (6.7%)
Healthy food is too expensive	104 (33.3%)	101 (32.4%)	52 (16.7%)	38 (12.2%)	17 (5.4%)
Embarrassed to exercise in public	78 (25.0%)	89 (28.5%)	63 (20.2%)	54 (17.3%)	28 (9.0%)
No access to safe places to exercise	61 (19.6%)	77 (24.7%)	81 (26.0%)	57 (18.3%)	36 (11.5%)
Lack of family support	56 (17.9%)	68 (21.8%)	74 (23.7%)	66 (21.2%)	48 (15.4%)
Lack of motivation	131 (42.0%)	88 (28.2%)	39 (12.5%)	34 (10.9%)	20 (6.4%)
Belief that weight loss is genetically impossible	84 (26.9%)	76 (24.4%)	61 (19.6%)	58 (18.6%)	33 (10.6%)
Insufficient guidance from physician	91 (29.2%)	85 (27.2%)	66 (21.2%)	44 (14.1%)	26 (8.3%)

Role of physicians and sources of information

A total of 138 (44.2%) participants reported that their primary care physician had discussed weight management with them during clinic visits (data not shown). The most common source of weight-related information was physician advice (102 participants [32.7%]), followed by social media (84 [26.9%]), friends or family (56 [17.9%]), and television or radio (29 [9.3%]) (Table 4). More than one-third of participants (107 [34.3%]) reported that they had received no structured advice about weight loss from any source.

**Table 4:** Patient willingness and motivators for lifestyle change (N=312) This table shows the willingness of 312 patients to participate in clinic-based structured weight loss programs and their most important motivating factors for lifestyle modification. Data are presented as number of patients (%).

Variable		No. of patients (%)
Willing to join clinic-based structured weight loss program	Yes	207 (66.3%)
	No	46 (14.7%)
	Not sure	59 (18.9%)
Most motivating factor for lifestyle change	Physician advice	96 (30.8%)
	Seeing results in others	64 (20.5%)
	Social support	52 (16.7%)
	Fear of health complications	71 (22.8%)
	Religious or moral beliefs	23 (7.4%)
	Other	6 (1.9%)

Factors associated with attempts to lose weight

In bivariate analyses (Table 5), women were more likely than men to report attempting to lose weight (147 of 199 women [73.9%] vs. 66 of 113 men [58.4%]; P=0.004). Participants with university or postgraduate education were also more likely to have attempted weight loss (139 of 181 [76.8%] vs. 74 of 131 [56.5%]; P<0.001). Attempts at weight loss were slightly more common among those with diabetes (104 of 142 [73.2%]) than those without (109 of 170 [64.1%]), though the difference was not statistically significant (P=0.08). There was no significant association between age group or employment status and weight loss attempts. Agreement with barriers such as lack of motivation and a physician not helping were similar between those who attempted and did not attempt weight loss (P>0.8 for both).

**Table 5:** Factors associated with attempting weight loss in the past year (N=312) This table shows bivariate associations between demographic, clinical, and barrier-related variables and whether patients attempted weight loss in the previous year. Data are presented as number of patients (%). P-values were calculated using the chi-square test for categorical variables. Statistical significance was determined at the 0.05 level (P < 0.05).

Variable		Attempted weight loss (n=213)	Did not attempt (n=99)	P-value
Sex	Female	147 (69.0%)	52 (52.5%)	0.004
	Male	66 (31.0%)	47 (47.5%)	
Age ≥50 years		68 (31.9%)	36 (36.4%)	0.43
University/postgraduate education		139 (65.3%)	42 (42.4%)	<0.001
Diabetes mellitus		104 (48.8%)	38 (38.4%)	0.08
Agreed: lack of motivation		108 (50.7%)	111 (88.9%)	0.84
Agreed: insufficient guidance from physician		98 (46.0%)	78 (78.8%)	0.93

Predictors of willingness to join a structured program

In multivariable logistic regression (Table 6), willingness to join a structured weight loss program was independently associated with female sex (adjusted odds ratio, 1.62; 95% CI, 1.01 to 2.60; P=0.045), higher educational level (adjusted odds ratio, 2.10; 95% CI, 1.28 to 3.46; P=0.003), and having identified a physician as a primary information source (adjusted odds ratio, 2.44; 95% CI, 1.47 to 4.06; P<0.001). Neither age group, employment status, reporting time constraints, or food costs as barriers were independently associated with willingness to participate in a structured program.

**Table 6:** Multivariable logistic regression: predictors of willingness to join a clinic-based weight loss program (N=312) This table presents adjusted ORs and 95% CIs from a multivariable logistic regression model assessing independent factors associated with willingness to participate in a structured weight loss program. ORs are adjusted for all variables listed. Statistical significance was determined at the 0.05 level (P < 0.05). OR, odds ratio; CI, confidence interval

Variable	Adjusted OR (95% CI)	P-value
Female sex	1.62 (1.01–2.60)	0.045
Age ≥ 50 years	0.74 (0.42–1.29)	0.28
University/postgraduate education	2.10 (1.28–3.46)	0.003
Agreed: “lack of time to exercise”	1.39 (0.84–2.29)	0.20
Agreed: “healthy food is expensive”	1.52 (0.91–2.55)	0.11
Physician was main info source	2.44 (1.47–4.06)	<0.001

## Discussion

In this cross-sectional study of overweight or obese adults attending family medicine clinics in Saudi Arabia, we found that fewer than half of the participants had received weight-related advice from their physician, and only one-third had access to structured guidance. Despite this, more than two-thirds had attempted weight loss in the preceding year, most often by reducing portion sizes or increasing physical activity. The most frequently reported barriers to lifestyle modification were lack of motivation, limited time, and the perceived high cost of healthy food. Female sex, higher education, and receiving physician-delivered information were independently associated with willingness to participate in a structured program.

Barriers to lifestyle change reported by participants, especially lack of motivation and perceived time constraints, mirror findings from global surveys, but cultural and socioeconomic nuances may influence their relative importance in this context. For example, the prominence of family or cultural obligations as a barrier may reflect gendered caregiving roles or social expectations specific to the region [14,15]. Additionally, cost-related concerns likely reflect disparities in access to affordable healthy food options, particularly in urban and lower-income communities [14,16].

The findings underscore the persistent gap between clinical guidelines for obesity management and the realities of primary care delivery in the region. Although international and local guidelines recommend routine counseling and referral to structured interventions for patients with obesity, our data suggest that these practices are not consistently implemented. This observation is consistent with prior reports from Middle Eastern countries, which have identified limited physician engagement in weight management as a systemic barrier [17]. The low frequency of counseling observed in our study may reflect competing clinical priorities, limited consultation time, or inadequate provider training in behavioral interventions.

Participants overwhelmingly reported reliance on non-medical sources, particularly social media, for weight-related information. While this may reflect the accessibility of digital platforms, it raises concerns about the accuracy and safety of information being consumed. Prior studies have shown that health-related content on social media is often unregulated and may promote ineffective or harmful practices [14-16]. The association between physician-delivered advice and willingness to engage in structured programs in our study highlights the potentially pivotal role of primary care in shifting patient behavior when appropriate counseling is provided.

The independent association between higher educational attainment and both weight loss attempts and willingness to participate in structured programs suggests that health literacy may be a critical determinant of engagement. Previous research has similarly demonstrated that individuals with higher education are more likely to perceive obesity as a modifiable condition and to adopt preventive behaviors [15,17]. Addressing this gap will require targeted, culturally adapted educational initiatives that improve understanding and empower patients with practical skills for change.

This study has several limitations. First, the cross-sectional design precludes causal inference. Second, self-reported data are subject to recall and social desirability biases, particularly regarding weight loss behaviors. Third, although participants were recruited from multiple clinics, the sample may not fully represent rural populations or those who do not seek primary care. Finally, our use of a structured multiple-choice questionnaire may have limited the depth of responses, particularly regarding complex behavioral factors.

## Conclusions

In this cross-sectional study of overweight or obese adults in Saudi family medicine clinics, we found that while most patients had attempted weight loss, a substantial proportion faced persistent barriers related to motivation, time, cost, and lack of structured support. Physician engagement and higher educational attainment were positively associated with willingness to pursue structured programs, underscoring the critical role of primary care in facilitating behavior change. These findings highlight the need for targeted, culturally sensitive strategies to support lifestyle modification and close the gap between clinical guidelines and everyday practice in obesity management.

## Appendices

**Table 7:** The questionnaire used in the study

Section	Question	Answer Choices
1. Demographics	What is your age?	Open-ended (years)
	What is your gender?	☐ Male ☐ Female
	What is your marital status?	☐ Single ☐ Married ☐ Divorced ☐ Widowed
	What is your highest level of education?	☐ No formal education ☐ Primary ☐ Secondary ☐ University ☐ Postgraduate
	What is your employment status?	☐ Employed ☐ Unemployed ☐ Student ☐ Retired
2. Clinical Profile	What is your weight?	Open-ended (kg)
	What is your height?	Open-ended (cm)
	How long have you had obesity?	☐ <1 year ☐ 1–5 years ☐ >5 years
	Do you have any of the following chronic diseases?	☐ Diabetes ☐ Hypertension ☐ Dyslipidemia ☐ Hypothyroidism ☐ None
3. Weight Loss Attempts	Have you tried to lose weight in the past 12 months?	☐ Yes ☐ No
	What methods did you use?	☐ Diet ☐ Exercise ☐ Medications ☐ Bariatric surgery ☐ Herbal remedies ☐ Others: _______
4. Sources of Information	Where do you mainly get information about weight loss?	☐ Physician ☐ Social media ☐ Friends/family ☐ TV/radio ☐ Online articles ☐ Books
5. Barriers to Lifestyle Modification	Please indicate your agreement with the following statements:	Likert Scale: ☐ Strongly Disagree ☐ Disagree ☐ Neutral ☐ Agree ☐ Strongly Agree
	I don't have time to prepare healthy meals or exercise.	Likert Scale: ☐ Strongly Disagree ☐ Disagree ☐ Neutral ☐ Agree ☐ Strongly Agree
	I lack motivation to lose weight.	Likert Scale: ☐ Strongly Disagree ☐ Disagree ☐ Neutral ☐ Agree ☐ Strongly Agree
	I don’t have family support.	Likert Scale: ☐ Strongly Disagree ☐ Disagree ☐ Neutral ☐ Agree ☐ Strongly Agree
	Healthy food is too expensive.	Likert Scale: ☐ Strongly Disagree ☐ Disagree ☐ Neutral ☐ Agree ☐ Strongly Agree
	I feel embarrassed to exercise in public.	Likert Scale: ☐ Strongly Disagree ☐ Disagree ☐ Neutral ☐ Agree ☐ Strongly Agree
	There are no safe places to exercise in my area.	Likert Scale: ☐ Strongly Disagree ☐ Disagree ☐ Neutral ☐ Agree ☐ Strongly Agree
	Obesity runs in my family and can't be changed.	Likert Scale: ☐ Strongly Disagree ☐ Disagree ☐ Neutral ☐ Agree ☐ Strongly Agree
	My physician did not advise me to lose weight.	Likert Scale: ☐ Strongly Disagree ☐ Disagree ☐ Neutral ☐ Agree ☐ Strongly Agree
6. Physician Involvement	Has a physician ever discussed your weight with you?	☐ Yes ☐ No
7. Willingness to Join Program	Are you willing to join a structured clinic-based weight loss program?	☐ Yes ☐ No ☐ Not sure
8. Motivators for Lifestyle Change	What would motivate you the most to lose weight?	☐ Physician’s advice ☐ Social support ☐ Seeing others succeed ☐ Fear of health consequences ☐ Religious/spiritual beliefs ☐ Other: _______

## References

[1] Almohammedsaleh AS, Alshawaf YY, Alqurayn AM (2024). Barriers to a healthy lifestyle among obese patients attending primary care clinics in Al-Ahsa, Saudi Arabia. Cureus.

[2] Al Saud LM, Altowairqi SE, Showail AA, Alzahrani BS, Arnous MM, Alsuhaibani RM (2024). Primary care physicians' knowledge and attitudes about obesity, adherence to treatment guidelines and its' association with confidence to treat obesity at the Saudi Ministry of Interior primary health care centers. J Family Med Prim Care.

[3] Caterson ID, Alfadda AA, Auerbach P (2019). Gaps to bridge: Misalignment between perception, reality and actions in obesity. Diabetes Obes Metab.

[4] Alfulayw MR, Almansour RA, Aljamri SK (2022). Factors contributing to noncompliance with diabetic medications and lifestyle modifications in patients with type 2 diabetes mellitus in the eastern province of Saudi Arabia: a cross-sectional study. Cureus.

[5] Carrasco D, Thulesius H, Jakobsson U, Memarian E (2022). Primary care physicians' knowledge and attitudes about obesity, adherence to treatment guidelines and association with confidence to treat obesity: a Swedish survey study. BMC Prim Care.

[6] Alageel S, Alhujaili M, Altwaijri Y, Bilal L, Alsukait R (2023). Barriers and facilitators to adopting healthier lifestyle among low-income women in Saudi Arabia: a qualitative study. Health Expect.

[7] Arabi H, Altaf LZ, Khashoggi AA, Alwazzan SB, Aldibasi O, Jamil SF (2022). Readiness to change among parents of overweight/obese children in Saudi Arabia and influencing factors. J Family Med Prim Care.

[8] Al-Ghamdi S, Shubair MM, Aldiab A (2018). Prevalence of overweight and obesity based on the body mass index; a cross-sectional study in Alkharj, Saudi Arabia. Lipids Health Dis.

[9] Kebbe M, Perez A, Buchholz A (2018). Barriers and enablers for adopting lifestyle behavior changes in adolescents with obesity: a multi-centre, qualitative study. PLoS One.

[10] Anand VV, Zhe EL, Chin YH (2023). Barriers and facilitators to engagement with a weight management intervention in Asian patients with overweight or obesity: a systematic review. Endocr Pract.

[11] Thomson RL, Buckley JD, Brinkworth GD (2016). Perceived exercise barriers are reduced and benefits are improved with lifestyle modification in overweight and obese women with polycystic ovary syndrome: a randomised controlled trial. BMC Womens Health.

[12] Burgess E, Hassmén P, Pumpa KL (2017). Determinants of adherence to lifestyle intervention in adults with obesity: a systematic review. Clin Obes.

[13] Helland MH, Nordbotten GL (2021). Dietary changes, motivators, and barriers affecting diet and physical activity among overweight and obese: a mixed methods approach. Int J Environ Res Public Health.

[14] Goldstein RF, Boyle JA, Lo C, Teede HJ, Harrison CL (2021). Facilitators and barriers to behaviour change within a lifestyle program for women with obesity to prevent excess gestational weight gain: a mixed methods evaluation. BMC Pregnancy Childbirth.

[15] Arora C, Malhotra A, Ranjan P, Vikram NK, Dwivedi SN, Singh N, Shalimar Shalimar (2021). Perceived barriers and facilitators for adherence to lifestyle prescription: perspective of obese patients with non alcoholic fatty liver disease from north India. Diabetes Metab Syndr.

[16] Cason-Wilkerson R, Goldberg S, Albright K, Allison M, Haemer M (2015). Factors influencing healthy lifestyle changes: a qualitative look at low-income families engaged in treatment for overweight children. Child Obes.

[17] Kreidieh D, Itani L, El Kassas G, El Masri D, Calugi S, Dalle Grave R, El Ghoch M (2018). Long-term lifestyle-modification programs for overweight and obesity management in the Arab states: systematic review and meta-analysis. Curr Diabetes Rev.

